# Assay for high-throughput screening of inhibitors of the ASC-PYD inflammasome core filament

**DOI:** 10.15698/cst2018.04.131

**Published:** 2018-03-21

**Authors:** Lorenzo Sborgi, Johanna Ude, Mathias S. Dick, Jonathan Vesin, Marc Chambon, Gerardo Turcatti, Petr Broz, Sebastian Hiller

**Affiliations:** 1 Biozentrum, University of Basel, Klingelbergstr. 70, 4056 Basel, Switzerland.; 2 Biomolecular Screening Facility, Ecole Polytechnique Fédérale de Lausanne (EPFL), Lausanne, Switzerland.

**Keywords:** high-throughput screening, polarization anisotropy assay, inflammasome, ASC, pyroptotic cell death, inflammation

## Abstract

The protein ASC is a central component of most inflammasome complexes, forming functional oligomeric filaments that activate large amounts of pro-caspase-1 for further IL-1β processing and the induction of Gasdermin D-dependent cell death. The central role of inflammasomes in the innate immune response pose them as new molecular targets for therapy of diverse acute, chronic and inherited autoinflammatory pathologies. In recent years, an increasing number of molecules were proposed to modulate inflammasome signalling by interacting with different components of inflammasome complexes. However, the difficult *in vitro* reconstitution of the inflammasome has limited the development of specific on-target biochemical assays for compound activity confirmation and for drug discovery in high throughput screening setups. Here we describe a homogeneous, pH-based ASC oligomerization assay that employs fluorescence anisotropy (FA) to monitor the *in vitro* filament formation of the PYD domain of human ASC. The absence of additional solubility tags as well as of proteolytic enzymes to initiate the filament reaction makes this assay suitable for testing the direct effect of small molecules on filament formation in high throughput format. The ability of the assay to detect modulators of filament formation was confirmed by using a non-filament forming PYD mutant. The high and reproducible Z’-factor of 0.7 allowed to screen 10,100 compounds by high-throughput screening (HTS) aiming to identify inhibitors of ASC filament. While none of these molecules was able to inhibit ASC filament formation *in vitro*, the assay is directly amenable to screen other compound classes or validate candidate molecules from other screens.

## INTRODUCTION

The inflammasome assembles as part of the innate immune response, initiated by the activation of different pattern recognition receptors (PRRs), such as certain NOD-like receptors (NLRs), AIM2-like receptors (ALRs) or Pyrin. These receptors have evolved to recognize conserved pathogenic molecular patterns or cellular stress associated molecules. The stimulation of PRRs and the consecutive activation of the inflammasome signalling pathways is essential to specify and amplify the cellular response required for host defence, tissue homeostasis and tumor immune surveillance in humans and other higher eukaryotes 1,2.

Inflammasomes are macromolecular complexes assembled in the cytosol of host cells, serving as activation platforms for the protease caspase-1 and subsequent pyroptotic cell death and pro-inflammatory cytokines release (e.g. IL-1β and IL-18) 3. The assembly of inflammasomes happens via homeotypic interactions between domains from the death-domain superfamily of the different components. Many of the PRRs thus recruit the adapter protein ASC (apoptosis-associated speck-like protein containing a caspase-recruitment domain (CARD)), which consists of two domains, the N-terminal pyrin domain (PYD) and the C-terminal CARD 4,5. Upon interaction with the activated receptors, ASC forms large oligomeric PYD filaments through homeotypic PYD-PYD interactions, displaying the CARD at the filament periphery. The CARD domain fulfils a dual role, on the one hand, it is required to interact with the CARD domain of pro-caspase-1, thus recruiting and activating it to caspase-1 6,7. On the other hand, the CARD domain condenses the speck via CARD-CARD interactions, influencing its macroscopic structure 6,7,8. While the isolated PYD domain is able to form elongated macroscopic specks, fully signalling-competent ASC inflammasomes require full-length ASC 6,7,8. Oligomerization of ASC is critical for inflammasome signalling, since it amplifies the signal produced by few activated receptor molecules by generating a multitude of sites for caspase-1 processing 8. Recently, the *in vitro* reconstitution of human and mouse ASC-PYD filaments and their structural description at high resolution, shed light on the molecular architecture and mechanism of assembly of this key element of the inflammasome complex 9,10.

While appropriate activation of inflammasome complexes represents the essential mechanism for pathogen clearance and host survival under physiological conditions, uncontrolled inflammasome activation is detrimental to host health. Aberrant activation of different NLR family members has been shown to cause rare auto-inflammatory disorders known as ‘inflammasopathies’ that are characterized by the increasing of production and secretion of IL-1β and IL-18 11,12,13,14. Remarkably, dysregulated activation of inflammasomes has been linked to a number of acquired auto-inflammatory diseases, such as gout, pseudogout, asbestosis, silica-mediated pulmonary disorder, atherosclerosis and type II diabetes mellitus 15,16,17.

Neutralization of the cytokine release by targeting inflammasome signalling thus offers considerable therapeutic promise to reduce detrimental inflammation in inflammasome-related disorders and inherited auto-inflammatory disorders. To date, approaches to therapeutically modulate the inflammasomes focused on the activation of NLRs and on molecules targeting the downstream signalling pathway such as caspase-1, IL-1β and IL-1-receptor 18,19,20. Alternatively, the possibility to block the ASC filament formation represents an additional option to tackle the inflammasome pathway therapeutically. In this sense, blocking the propagation of the filament at the level of PYD domain interactions of ASC would offer the advantage of the complete inhibition of caspase-1 recruitment independently from NLR activation. The possibility to uniformly block caspase-1 activation bypassing the specificity of NLRs might represent a powerful strategy to modulate cytokine release. Recently, a single domain antibody fragment that recognizes the CARD domain of ASC has shown the effectiveness to impair the ASC speck formation without affecting PYD functionality 21.

However, the possibility to inhibit the PYD domain interaction of ASC by means of small molecules has not been explored thus far. Previous attempts to target inflammasome proteins using biochemical assays have been challenging due to general low yield of protein expression and the intrinsic tendency of the components to form aggregates 22. The recent identification of novel molecular key players of the inflammasome pathway and the development of functional protocols for inflammasome reconstitution *in vitro* opened the possibility to develop biochemical assays for high throughput screening of small molecule inhibitors.

Toward this direction, here we develop a fluorescence polarization-based assay capable of monitoring PYD filament formation *in vitro* without the interference of additional molecules in solution and thus suitable for high throughput screening of compounds. We optimized the runtime parameters and validated the functionality of our assay using a non-filament forming PYD mutant. Finally, we screened a library of 10,100 small molecule compounds.

## RESULTS 

**Figure 1:**
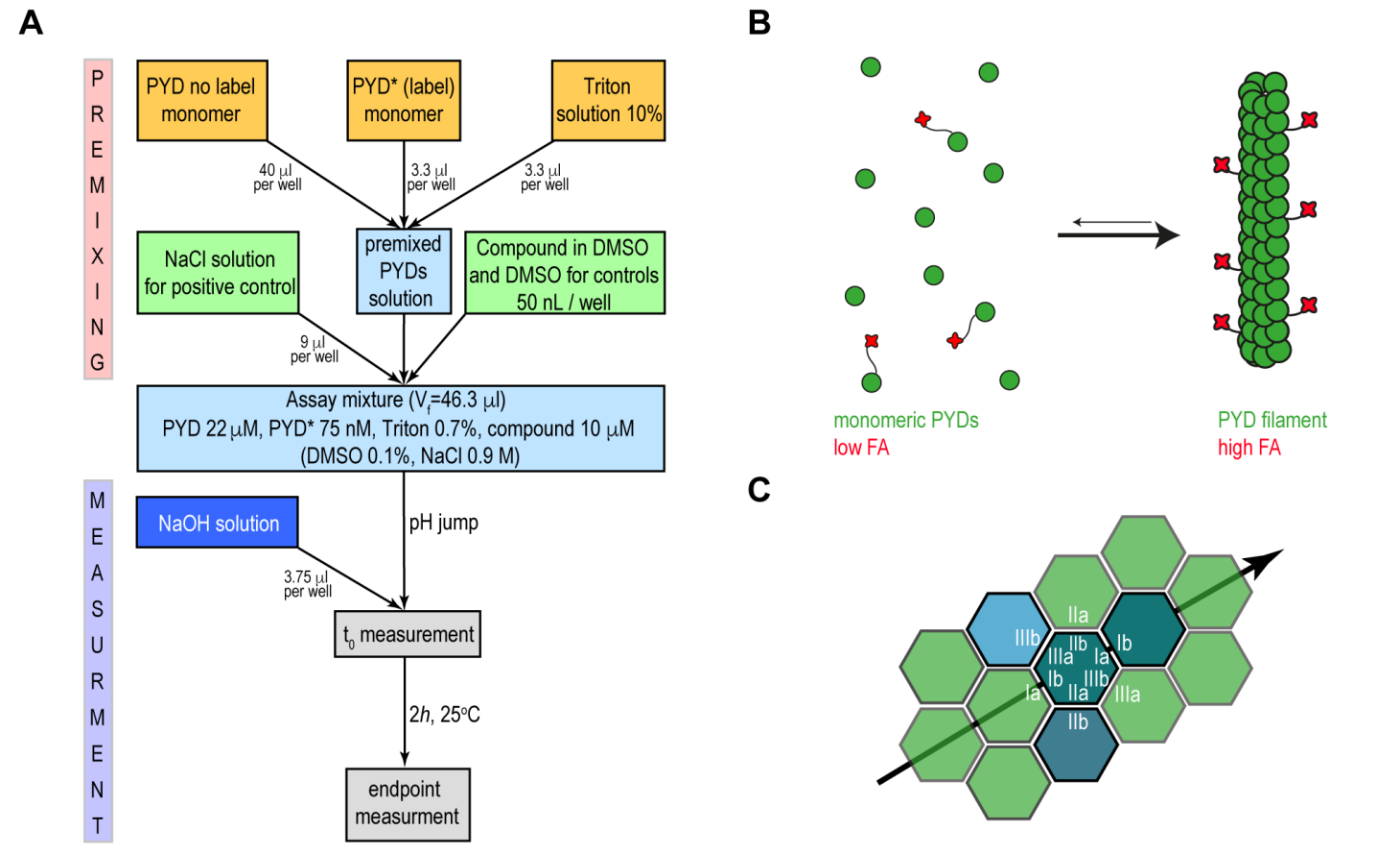
FIGURE 1: Principle of the high-throughput fluorescence anisotropy assay. **(A)** Flowchart of the assay procedure for the high-throughput screening. The initial PYD solution shown in light blue is prepared by mixing the specified volume amounts from 25 μM and 1 μM stock solutions of not labelled PYD and Fluorophore-labelled PYD (PYD*) respectively and 10% Triton solution, yellow rectangles. In the premixed solution labelled and unlabelled PYD are maintained in a soluble and monomeric forms by low pH condition. The addition to premixed PYD solutions of either NaCl as positive control or DMSO solution with and without the compounds are identified by the green rectangles. The resulting assay mixture is highlighted in light blue. The jump from acidic to physiological pH condition is achieved by addition of a fixed volume amount of a 60 mM NaOH solution to the assay mixture, highlighted by the dark blue rectangle. Fluorescence anisotropy is monitored as a single point measurement after incubation for 2 h at 25^°^C and filament formation is evaluated as the difference between fluorescence anisotropy at the initial and endpoint of the measurement, grey rectangles. **(B)** Scheme of the ASC filament formation during the fluorescence anisotropy (FA) assay. Monomeric PYD domain of ASC protein (green) and PYD* labelled with Alexa Dylight 488 fluorophore (red). The pH shift-induced filament formation leads to the anisotropy increases. **(C)** A schematic 2D diagram of the PYD filament arrangement where individual monomeric PYD domains required for filament formation are coloured in dark green, teal and light blue over the filament scaffold reported in light green. The asymmetric interfaces type I, II and III as observed on the human (PDB 3J63) and mouse (PDB 2N1F) PYD filament structures are labelled in white. The black arrow highlights the directionality of filament propagation within a single layer through the interactions between the interfaces type Ia and Ib.

Our approach aimed at developing a fluorescence anisotropy (FA)-based assay that recapitulates ASC filament formation with sufficient robustness to be used in high throughput format for inhibitor screening. Motivated by previous reports that fluorescence polarization-based experiments can monitor polymerization of ASC triggered by proteolytic digestion, 9 we developed an assay to specifically monitor the pH shift-induced PYD filament formation *in vitro*. The presence of solubility tags or an activation enzyme may well interfere with screening suitability and is therefore not preferred for high throughput assays. Recombinant wild type PYD from human ASC was expressed and purified from *E. coli *cells, with scalable yields in the high milligram range, following previously established protocols 10,23. For the production of fluorescence dye labelled PYD (PYD*), the maleimide-activated Dylight Fluor 488 was conjugated irreversibly to a single cysteine residue in an overnight reaction and under denaturing conditions (Supplementary Fig. 1A-C). Homogeneously purified wild type PYD and PYD* are maintained soluble and monomeric in low pH solution. Subsequently, a pH jump to physiological condition restores the ability of the PYD domain to polymerize into well-defined filaments (**Fig. 1A**). The transition from the monomeric to the filamentous form at physiological conditions results in a drastic change in the molecular rotational correlation time of PYD domains, which directly leads to a significant change in fluorescence anisotropy of the conjugated fluorophore of PYD* (**Fig. 1B**). The filament formation is monitored during time course FA experiments performed in 384-well plates, where the compound effect is evaluated relative to positive and negative controls.

This assay bases on a large experience with ASC and ASC-PYD filamentation, where we have observed filament formation under different protocols and conditions 8,10. The filaments can form either by slow or rapid dilution of a chaotrope-denatured state of ASC, or by slow or rapid dilution of an acidic protein solution to neutral or basic pH. The resulting filaments were monitored in numerous occasions by transmission and/or cryo-electron microscopy, as well as by dynamic light scattering 8,10. In none of the cases, we have observed unspecific aggregates of ASC-PYD, but only linear filaments in different lengths and concentrations (density), depending on the respective protocol. We thus find it safe to assume that in our controlled assay under specific conditions, filament growth is robust and the FA measurements are not affected by non-specific protein aggregation.

Recently, the *in vitro* reconstitution and characterization of human and mouse PYD filaments of ASC allowed a deeper understanding of the structural features and the specific interactions required for filament stability. The filament form of human and mouse PYD is characterized by a triple-stranded, right-handed helical filament in which each PYD interacts with six adjacent subunits through three asymmetric interfaces, types I-III (**Fig. 1C**) 9,10. A specific network of amino acid interactions is required for filament propagation: Interface type I involves interactions between residues of opposite charges while type II and III are defined by interactions involving both polar and hydrophobic side chains. The study of the effects of single amino acid mutations on the kinetics of filament formation and on the downstream cell signalling highlighted the K21A substitution from interface type I as a single point mutation capable to impair PYD filament formation and to abolish cytokines production 8,9. The identification of critical amino acids for filament formation emphasizes the opportunity to effectively block the filament propagation by targeting specific ‘hot spots’ on PYD surface by small molecules.

**Figure 2:**
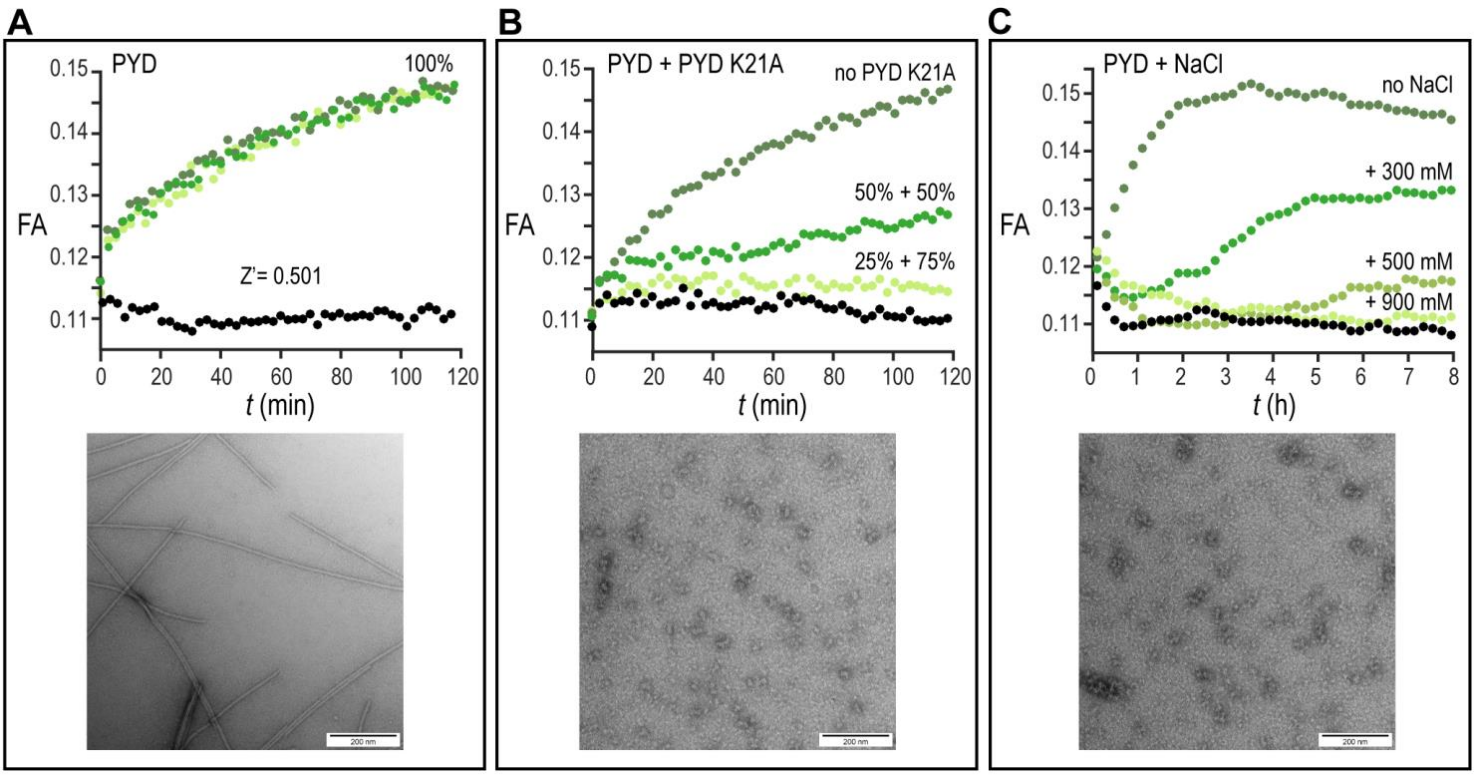
FIGURE 2: Proof of concept of the high-throughput fluorescence anisotropy assay. **(A-C)** The formation of the PYD filaments is monitored for three different conditions by the pH-based fluorescence anisotropy assay and negative-stained electron microscopy. **(A)** (*Upper*) Time-dependent fluorescence anisotropy (FA) measurements are reported for three independent experiments (shades of green) and for monomeric PYD without pH jump (black). Data points are recorded in 3 min intervals for a total time of 120 min. (*Lower*) EM image of negatively stained preparation of PYD filament after time-dependent fluorescence anisotropy measurement. (Scale bars, 200 nm). **(B)** (*Upper*) Time-dependent fluorescence anisotropy (FA) measurements for solutions of 100% PYD after pH jump (dark green), 50% PYD and 50% PYD K21A after pH jump (green), 25% PYD and 75% PYD K21A after pH jump (light green) and for monomeric PYD without pH jump (black). Data points are recorded in 3 min intervals for a total time of 120 min. (*Lower*) EM images of negatively stained preparation of 100% PYD K21A solution after incubation as in A. (Scale bars, 200 nm). **(C)** (*Upper*) Time-dependent fluorescence anisotropy (FA) measurements for solutions of PYD after pH jump (dark green), PYD with 300 mM NaCl after pH jump (green), PYD with 500 mM NaCl after pH jump (pale green), PYD with 900 mM NaCl after pH jump (light green) and for monomeric PYD without pH jump (black). Data are reported in 12 min intervals for a total time of 8 h. (*Lower*) EM image of negatively stained preparation of PYD solution with 900 mM NaCl after time-dependent fluorescence anisotropy measurement. (Scale bars, 200 nm).

We firstly evaluated the reproducibility of our assay by manually transferring 50 μl of assay mixture after pH jump directly on a 384-well plate. The kinetics of the filament formation monitored by FA time course experiments was highly reproducible and showed a sufficient Z’-factor = 0.5 (**Fig. 2A**). Since no specific small molecule inhibitors of ASC filament have been described so far, the reduction of filament formation was validated by measuring the concentration-dependent inhibitory effect of the non-filament forming PYD K21A mutant on wild type filament formation. For this experiment, 12.5 μM or 16.7 μM PYD K21A were mixed with 12.5 μM or 8.3 μM wt PYD, respectively, to a constant total protein concentration of 25 μM, and the fluorescence anisotropy was measured during time course experiments. The result shows that the lag-time, the filament growth rate and the final amount of formed filament were affected by PYD K21A, which thus inhibited filament formation with efficacies in the low micromolar range (**Fig. 2B**). A second possibility to inhibit filament formation biochemically is the use of high ionic strength. According to the observation that charged interactions from residues of interface type I play a central role for filament propagation, PYD polymerization is inhibited by addition of increasing salt concentrations. A concentration of 0.9 M NaCl was found sufficient for complete filament inhibition (**Fig. 2C**). All conditions tested in the pH shift-induced oligomerization assay have been validated in 384-well plate format by blotting protein solution on EM-grids after the assay execution. In agreement with the FA experiments, negatively stained images acquired with transmission electron microscopy confirm the filament formation for the wild type PYD and absence of filaments in the PYD K21A mutant and for wild type PYD in presence of 0.9 M NaCl, (**Fig. 2A-C**).

**Figure 3:**
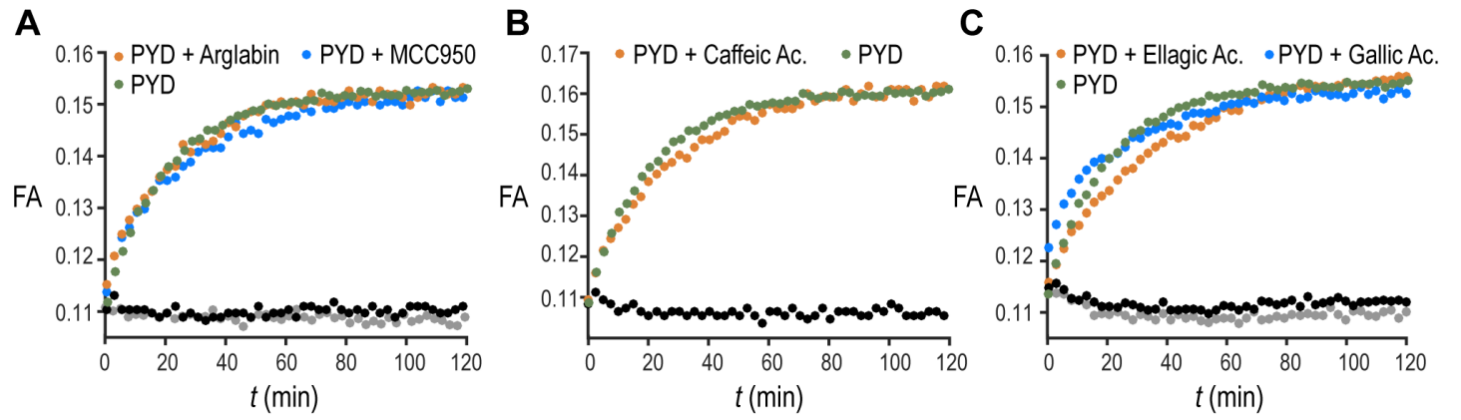
FIGURE 3: Inability of five small molecules to inhibit PYD filament formation. **(A-C) **Time-dependent fluorescence anisotropy (FA) measurements after pH jump on PYD only solution (dark green) and on PYD solution premixed with 20 μM final concentration of **(A)** Arglabin (orange) or MCC950 (light blue), **(B)** Caffeic acid phenethyl ester (orange), **(C)** Ellagic acid (orange) or Gallic acid (light blue). Time-dependent fluorescence anisotropy (FA) measurements for PYD incubated with compounds without pH jump are shown (black, gray). Data are reported in 3 min intervals for a total time of 120 min.

The validation of our assay using a non-filament forming PYD K21A mutant confirms our biochemical assay as a suitable tool to recapitulate the features of ASC filament formation and thus extends it to screening of small molecules. An increasing number of compounds are proposed to inhibit inflammasomes 24. Out of this list, we tested the effect of five different molecules reported in literature to inhibit IL-1β production by direct or indirect reduction of ASC filament formation (**Fig. 3A-C**). The natural product Arglabin and the compound MCC950 have been shown to inhibit specifically the NLRP3 receptor activation and thus significantly reduce ASC speck formation 25,26,27, while caffeic acid phenethyl ester was suggested to effect ASC filament formation by inhibiting ASC phosphorylation 28. In addition, we decided to test the effect of the flavonoid ellagic acid, which is chemically highly similar to the flavonoid Quercetion, which has been suggested to be ASC inflammasome inhibitors in cells 29. All these molecules, when tested in our *in vitro* assay at a concentration of 20 μM, showed no inhibition or enhancement of the ASC PYD filament formation (**Fig. 3A-C**), in agreement with the reported specificity of Arglabin and MCC950 for the NLRP3 receptor, and the suggested action of Quercetin as a kinase inhibitor 29. The absence of a detectable effect of caffeic acid phenethyl ester could suggest that it may exhibit its inhibitory effect on full-length ASC by interfering with the CARD domain of ASC, which was not present in our assay.

**Figure 4:**
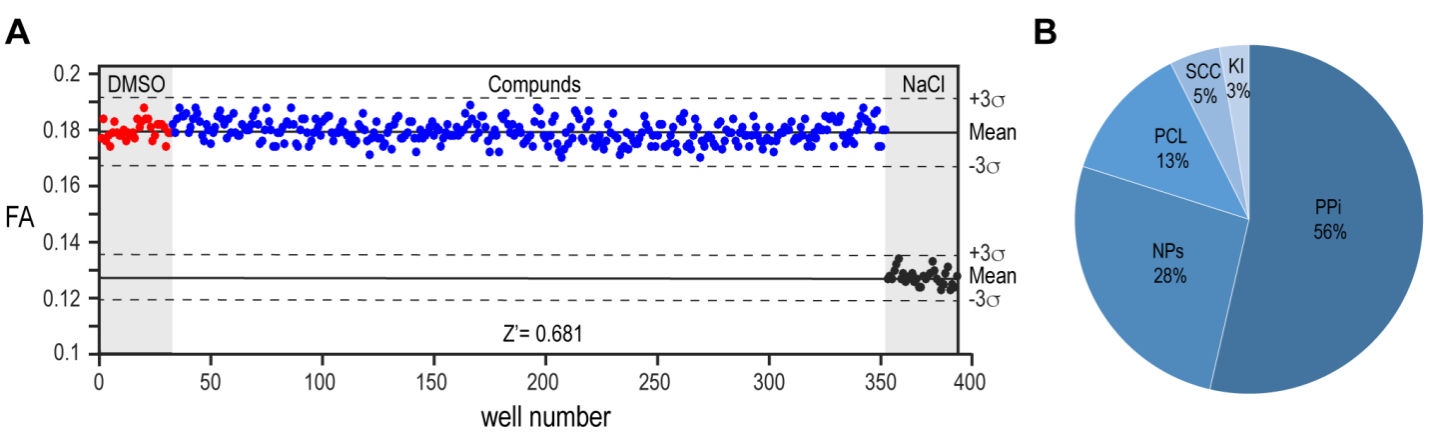
FIGURE 4: High-throughput screening of compound library. **(A)** Evaluation and visualization of fluorescence anisotropy data from the high-throughput screening of 10,100 compounds. The screening is performed on 384-well plates where the first and the last two columns are used for negative controls reported in red (0.1% DMSO; no drug added) and positive controls reported in black (900 mM NaCl). The areas used for positive and negative controls are highlighted in light grey. The middle columns (320 wells) are used to test compounds (10 μM compound final concentration, final DMSO concentration 0.1%) and reported in blue. The library was processed in duplicate on 64 plates. The Z’-factor determination for plate validation is calculated using the fluorescence anisotropy signals of the controls and compounds wells. The solid lines represent the mean fluorescence anisotropy value for controls and compounds measurements, the dashed lines denote the average ± 3 standard deviation of the negative and the positive controls, respectively. In this example, a Z’-factor of 0.681 is suitable to validate the plate. **(B)** Pie chart of 10,100 compounds from BSF-ACCESS Chemical Collection. Five different classes of compounds are screened: PPi (Protein-Protein interaction, 5’411 compounds), NPs (Natural Products, 2’654 compounds), PCL (The Prestwick Chemical Library, 1’280 compounds), SCC (Swiss Chemical Collection, 474 compounds) and Kinase Inhibitors, 275 compounds. The relative percentage of each class of compounds over the entire screened library is reported.

The implementation of our assay into high throughput screening format required some further optimization. The homogenous preparation of large batches of proteins and the use of automated dispenser to aliquot each solution maximized the assay response as demonstrated by a Z’-factor of >0.7 calculated from the control wells on each assayed plate (**Fig. 4A**). We proceeded with the screening of 10,100 compounds from different sub-libraries in order to cover a wide range of chemical and structural diversity (**Fig. 4B**). For primary screening execution, compounds were aliquoted at a final concentration of 10 μM and the FA was measured in each well as an end point measurement at a time point of 2 hours after the jump to physiologic pH condition. The high throughput screening (HTS) scores of each compound were evaluated relative to the average positive and negative controls signals. After performing screening assays in duplicates and the removal of autofluorescent compounds interfering with signal detection, six molecules out of the entire library emerged as primary potential hits from screening execution (**Supplementary Fig. 2A**). The four non-cytotoxic compounds from this shortlist showed a clear concentration-dependent effect that could be validated by independent dose response assay. However, the observed concentration-dependent effect on the interference assay identified all the molecules as false positives (**Supplementary Fig. 2B, C**). While our biochemical assay is thus shown to be of good value in assessing the selectivity of PYD filament modulators, the lack of positive hits from high throughput screening campaign highlights the PYD domain as a challenging protein to be targeted by small molecules.

## DISCUSSION

Here, we developed an on-target biochemical assay designed to specifically identify compounds that directly block or enhance the oligomerization of the PYD domain of human ASC. The assay is simple to be executed and the production of single components is scalable to support high throughput screening formats. The triggering of filament formation is based on restoring physiological pH starting from an acidic PYD solution where ASC-PYD is maintained soluble and monomeric. Upon the jump to neutral pH condition, the filament formation is initiated and monitored using FA time course experiments. This approach makes the assay suitable for direct and specific detection of molecules that modulate PYD filament formation and overcomes possible interferences arising from the presence of additional solubility tags coupled to the PYD domain and enzymes used to start filament formation. The suitability for high throughput screening was proved by evaluating the inhibitory effect of the PYD K21A mutant in a concentration-dependent manner. As result, the pH-induced fluorescence anisotropy-based experiment is a sensitive tool able to recapitulate the characteristic features of the ASC filament formation and it is sufficiently robust for high throughput screening implementation.

The assay was used to screen 10,100 compounds with diverse chemical and structural properties with the aim to find inhibitors of PYD filament formation. The ability to block protein-protein interactions by small molecules is related to the shape and composition of the interaction interface. The suitability of a small molecule to target a protein-protein interface depends on several conditions including the availability of appropriate types of amino acids, as well as the shape and the location of the interface on the protein surface. The fulfilment of these conditions can make the discovery of suitable inhibitors challenging. On the other hand, the formation of PYD filaments is a finely tuned mechanism driven by multiple stabilizing forces distributed between three different interfaces. Tight interactions of amino acids at the interface type I play a fundamental role in filament propagation as confirmed by the effect of single point mutations and by the inhibition at high salt concentration. Residues from this interface seem to represent the crucial ‘hot spot’ for the recognition of PYD domains and thus effective for filament inhibition by the interaction with competitive small molecules. However, the lack of positive hits from our screening activity suggests that the binding interfaces of PYD domain might represent difficult or intractable targets for small molecules. This might be due to a relatively flat interacting surface area and to the lack of an obvious binding cleft on the PYD domain surface. Our assay provides a platform for further intensive screening approaches, perhaps including other classes of inhibitor candidates, such as cyclic peptides.

## MATERIALS AND METHODS

### Cloning, Expression, and Purification of wild type and fluorescence labelled PYD domain of ASC

cDNA coding for the PYD domain of human ASC protein (residues 1-91) was cloned with a C-terminal six-histidine tag into a pET28a vector under the control of a T7 promoter. A GSGSLE linker was introduced at the C terminus to minimize the His-tag effect on protein structure. For engineering the fluorescence dye labelled PYD domain of ASC (PYD*), an additional point mutation E97C was introduced on the C-terminal linker. Both protein constructs were transformed in BL21(DE3) *E. coli* strains, and the proteins were expressed by growing the cultures at 37°C to an OD_600_ of 0.8 followed by induction with 1 mM isopropyl β-d-1-thiogalactopyranoside for 4 h. For the purpose of high throughput screening 80 and 5 liters of LB medium culture were grown for expression of PYD and PYD(E97C) of ASC, respectively. The cells were harvested by centrifugation and the pellet was resuspended in 50 mM phosphate buffer (pH 7.5), 300 mM NaCl, 0.1 mM protease inhibitor. The resuspended cells were incubated for 1 h at room temperature with DNase I and then were disrupted by high‐pressure microfluidization on ice and centrifuged at 20,000 × *g* at 4°C for 30 min. The inclusion body pellet containing both proteins was resuspended in 50 mM phosphate buffer (pH 7.5), 300 mM NaCl, 6 M guanidinium hydrochloride and was centrifuged at 20,000 × *g* at 4°C for 30 min. The supernatant was incubated for 2 h at room temperature with preequilibrated Ni-NTA affinity resin (Thermo Scientific) and then was passed through a column for gravity flow purification. The column was washed with 20 column volumes of resuspension buffer containing 20 mM imidazole, and the fusion protein was eluted with three column volumes of the same buffer containing 500 mM imidazole. For the purification of PYD(E97C), all purification steps were carried out at 4°C and 2 mM DTT was added to all buffers. For labelling of PYD(E97C) with maleimide-activated DyLight Fluor 488 dye (Thermo Scientific), the elution fraction from Ni-NTA affinity column exchanged via a PD‐10 column (GE Healthcare) into 50 mM phosphate buffer (pH 7), 150 mM NaCl, 8 M Urea, 1 mM EDTA. The labelling reaction proceeded by incubating overnight with 5-fold excess of dye at 25°C under moderate stirring condition and UV-light protection. The labelling efficiency of the reaction was found to be around 50%. Control experiments showed that the presence of unlabelled protein did not significantly influence filament formation kinetics and that no detectable preformed dimers were present in the PYD* sample (Supplementary Fig. 1B). The precipitated fraction of the protein was removed by centrifugation at 20,000 × *g* at 4°C for 15 min and the supernatant fraction was used for the subsequent refolding procedure (Supplementary Fig. 1C). Refolding of PYD and PYD* domain of ASC was performed by decreasing the pH to 3.7 and dialysis against 50 mM glycine buffer (pH 3.7), 150 mM NaCl. The proteins were further purified to monomeric soluble forms on a preequilibrated Superdex 75 gel filtration column (GE Healthcare). The measurement of PYD* stock concentration is calculated as subtraction of the absorbance contribution of the dye at 280 nm from the protein absorbance at 280 nm. Stocks of 25 μM and 1 μM of PYD and PYD* respectively were prepared and used immediately or frozen in aliquots using liquid N_2_.

### Transmission Electron microscopy

For negative staining electron microscopy of the PYD filament of ASC, 2 μl of the sample after time-dependent fluorescence anisotropy measurements were placed on glow-discharged copper grids and stained with 5% uranyl acetate for 3 min and air-dried. Images were acquired using a Tecnai G2 spirit 120kV Transmission Electron Microscope (TEM) with a LaB6 filament and an EMSIS MORADA camera.

### Fluorescence polarization screening assay

Preliminary studies for the high throughput screening assay development, optimization and validation were performed directly on 384-well plate (Black Polystyrene, Non-Binding Surface, F-bottom, Low flange, Corning 3575) format required for the execution of high throughput screening. The optimal condition of the assay mixture consists of 20 μM PYD, 70 nM PYD *, 0.7% Triton, pH = 3.7. The use of instantly defrosted aliquots of PYD solution required the additional steps of centrifugation at 20,000 × g at 4°C for 20 min and filtration with disposable syringe filter units of 0.1 mm pore size (Sigma Aldrich) to remove possible filament seeds. At low pH, the PYD domain is maintained in a soluble and monomeric state. Upon the manual addition 3.4 μl of 60 mM NaOH solution, pH = 7 is reached for a total reaction volume of 50 μl per well. The transition from monomeric proteins to filaments results in a drastic change in the molecular rotational correlation time, which directly leads to a significant change in fluorescence anisotropy (FA) of the conjugated fluorophore. The FA time course experiments were performed on Synergy H1 Hybrid microplate reader from Biotek using excitation at 485 nm and emission at 528 nm. An initial linear shake was set for 10 s and data was acquired in 10 s intervals for a total time of 120 min. The kinetics of the filament formation were monitored by the comparison of the time-dependent fluorescence anisotropy changes of the PYD domain with and without pH jump. The reduction of filament formation upon small molecule interaction was simulated by measuring the concentration-dependent inhibitory effect of PYD K21A mutant on wild type filament formation. For this experiment, 12.5 or 16.7 μM of PYD K21A were mixed with 12.5 or 8.3 μM of wild type PYD respectively, and fluorescence anisotropy was measured during time course experiments. Comparable inhibitory effect on the filament formation was achieved by increasing concentrations of NaCl added to the final premixed solution. For this purpose, 300, 500 and 900 mM NaCl were added independently to premixed PYD solutions and fluorescence anisotropy was acquired in 3 min intervals for a total time of 8 h. The replacement of the ASC-PYD K21A with 900 mM NaCl as positive control helped to simplify the execution of the high throughput screening assay.

### Z’-factor determination 

The assay quality for the different experimental setups in 384-well format was estimated by determining the Z’-factor using NaCl as a positive control and DMSO as a negative control. The Z’-factor was calculated according to the formula 30:

**Formula 1:**
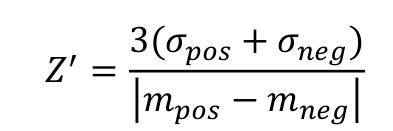


where σ and *m* are the standard deviation and the average values, respectively, of the fluorescence anisotropy signals of the negative and positive control wells. For final optimization and quality evaluation of the HTS assays, the Z’-plates (in 384-well format) were prepared using half the plate as a negative control (DMSO, 0.1%) and the other half as a positive control (900 mM NaCl with DMSO, 0.1%). The Z’-factor was also determined for every plate relative to the negative and positive controls, first and last columns of the plates respectively, in order to verify the screening assay quality and validate each plate of the screen.

### Compound libraries

A total of 10,100 chemical compounds were selected from a set of five different libraries: The protein-protein interaction library (PPi, 5’411 compounds, Life Chemicals); the natural products library (NP, 2’654 compounds, Analyticon-InterBioscreen); the Prestwick chemical library (PCL, 1’280 compounds, Prestwick Chemicals); the Swiss Chemical Collection (SCC, 474 compounds, in-house library); and a kinase inhibitor library (Ki, 275 compounds, SelleckChem and Enzo Life Sciences). All molecules are supplied as 10 mM stock solutions in DMSO. Compounds were stored in the dark at -20°C under dry air using an automated storage system (A3-RTS store from Brooks) and their chemical integrity was controlled regularly by RP-HPLC, coupled to ESI-MS and CAD detector (Thermo).

### Primary high throughput screening assay protocol

The selected 10,100 compounds were dispensed into 32 barcoded 384-well plates, using an acoustic liquid handler Echo 550 (Labcyte Inc. Sunnyvale, CA). Each drug was added once (one well per compound), at a volume of 50 nl, yielding a final compound concentration of 10 μM and a final DMSO concentration of 0.1%. The first two columns of each plate were used as negative control (no compound added) and filled with an equivalent volume of DMSO (50 nl/well) while the last two columns were filled with 9 μl NaCl (final concentration of 900 mM) for the positive control. The optimal assay mixture condition for ASC filament formation described above was scaled-up to a final bulk volume of 16 ml required to perform the remaining 320 single-well reactions per plate in presence of the compounds. The freshly prepared mixture solution of 22 μM PYD, 75 nM of PYD*, 0.7% Triton, pH = 3.7 was filtered and dispensed as 46 μl volume to each well of the plate. The automated dispenser (Multiflow from Biotek) was previously primed with glycine buffer pH=3.7 to avoid undesired pH changes of the PYD solution. The addition 3.4 μl of 60 mM NaOH solution was performed using the liquid handling workstation Caliper Sciclone and three times mixing of solution is performed on final reaction volume. PYD filament formation was evaluated as the difference in FA between the pH jump and the ending point of 120 min. The FA measurements were performed on a Tecan Infinite F500 microplate reader using excitation at 485 nm and emission at 530 nm. The data sets were evaluated as HTS scores, where a value of about 0.12 was assigned to the average FA of the positive control wells and a value of about 0.18 to the negative control wells. Screened compounds with HTS scores lower than the average of the negative controls - 3 times σ were classified as ‘Hit compounds’. An in-house Laboratory Information Management System (LIMS) was used for basic data processing, management, visualization and statistical hit validation. The screening of the compounds was performed in duplicate and molecule scores were calculated as average ± standard deviation.

### Dose response and interference studies 

The resulting selected compounds from the primary screening assay were further tested for their activity in a dose-response manner in 384-well plates using the FA assay. Compound dilution series were generated using an Echo 550 dispenser. Compounds were tested at 8 different concentrations (μM): 100, 57.1, 32.7, 18.7, 10.7, 6.1, 3.5, 2.0 with 2 replicates per concentration. The background fluorescence that might interfere with FA readout was evaluated testing the compounds alone by FA under the same conditions.

## SUPPLEMENTAL MATERIAL

Click here for supplemental data file.

All supplemental data for this article are also available online at http://www.cell-stress.com/researcharticles/assay-for-high-throughput-screening-of-inhibitors-of-the-asc-pyd-inflammasome-core-filament/.

## Abbreviations

ASC: apoptosis-associated speck-like protein containing a CARD
CARD: caspase recruitment domain
FA: fluorescence anisotropy
NLR: NOD-like receptor
PRR: pattern recognition receptor
PYD: pyrin domain
