# Supplementary material for: Assay for high-throughput screening of inhibitors of the ASC-PYD inflammasome core filament
Source: Cell Stress. 2018 Mar 21;2(4):82–90. doi: 10.15698/cst2018.04.131 (PMC6551747; doi:10.15698/cst2018.04.131)
Supplement: Supplementary file 1 [file ces-02-082-s01.pdf]

SUPPLEMENTARY MATERIAL

Assay for high-throughput screening of inhibitors of the ASC-PYD inflammasome core filament

Lorenzo Sborgi, Johanna Ude, Mathias S. Dick, Jonathan Vesin, Marc Chambon, Gerardo Turcatti, Petr Broz, Sebastian Hiller

Content: Supplementary Figures S1, S2

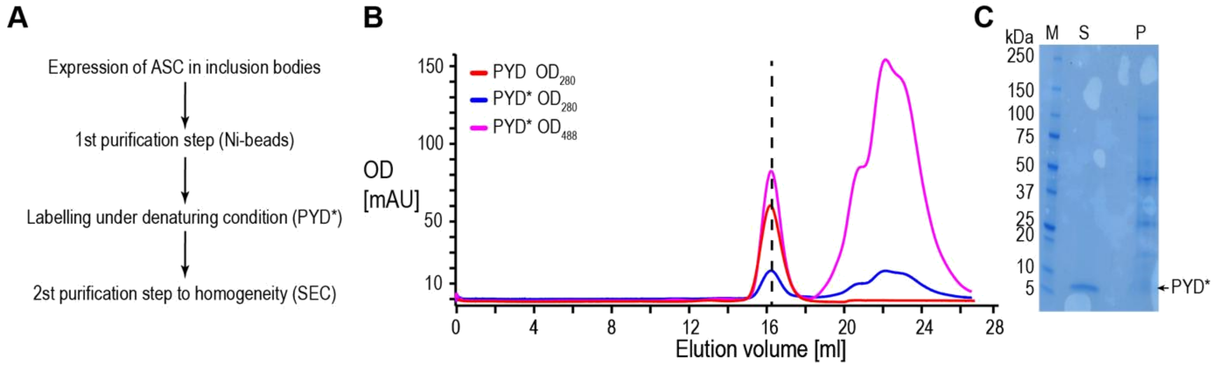

Supplementary Figure 1. Characterization of ASC PYD in solution. A) Scheme of PYD* labelling procedure with Maleimide-activated DyLight Fluor 488 dye. The PYD(E97C) domain of ASC is expressed in inclusion bodies. After a first step of purification under denaturing condition using a Ni-NTA affinity resin the Maleimide-activated Dylight Fluor 488 is conjugated irreversibly to the single cysteine residue on ASC PYD in an overnight reaction. A final size exclusion step of purification leads to the required protein homogeneity. **B)** Gel filtration profiles of PYD domains of ASC protein and PYD* using 50 mM glycine buffer (pH 3.7), 150 mM NaCl. Recombinant wild-type and labelled PYD domain of ASC eluted as monomeric fractions. **C)** Purity of PYD samples monitored by Coomassie-stained SDS-PAGE. Lane S: Recombinant PYD* sample after gel filtration purification. Lane P: Protein pellet occurring during incubating of PYD overnight with 5-fold excess of DyLight Fluor 488 dye.

A

Library	CatalogID	Name	Score	ScoreSD
PCL	Prestw-1196	Topotecan	0.26	0.060
PPI	0526-1202	F0526-1202	0.37	0.026
SCC	IH029	4,8-bis(3-hydroxypropyl)-12-octyl-8,12-dihydrobenzo[8,1][2,7]naphthyridino [3,4,5,6-klmn]acridin-3a2(4H)-ylium tetrafluoborate	1.59	0.025
SCC	IH031	4,8,12-tris(3-hydroxypropyl)-8,12-dihydrobenzo[8,1][2,7] naphthyridino[3,4,5,6-klmn]acridin-3a2(4H)-ylium tetrafluoroborate	1.38	0.12
SCC	WA329d	9-(2,6-dimethoxyphenyl)-10-hydroxy-1,8-dimethoxy-9,10-dihydroacridin-9-ylium tetrafluoroborate	1.00	0.0096
SCC	WA36a	4,8,12-tripropyl-8,12-dihydro-4H-benzo[1,8][2,7]naphthyridino[3,4,5,6-klmn]acridin-3a2-ylium tetrafluoroborate	0.72	0.13

B

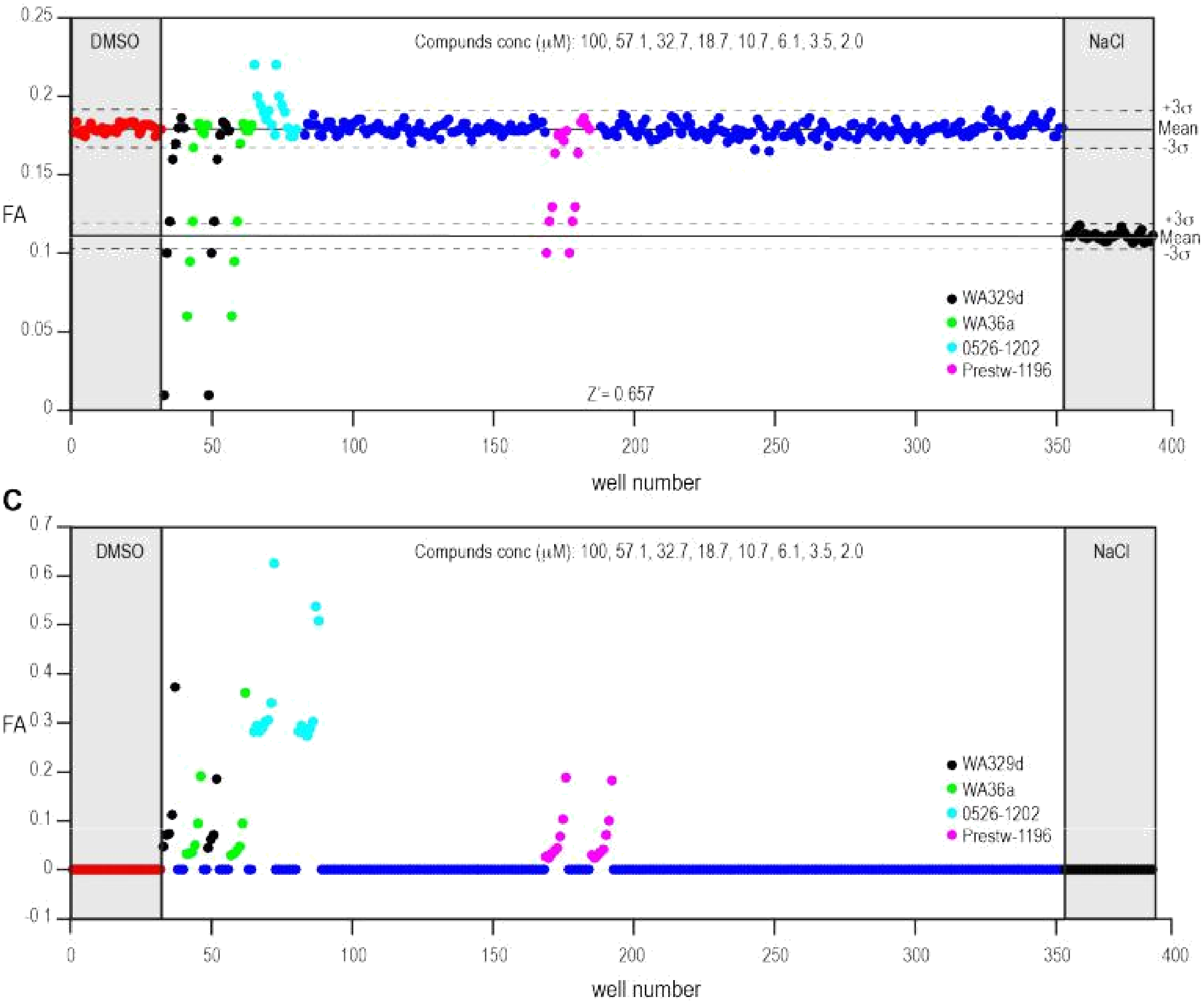

Supplementary Figure 2. Results from high throughput screening. A) Summary of the six potential hits from primary screening. The screening of the compounds was performed in duplicate and the normalized scores were calculated as average ± standard deviation (SD). The Z' factors are above 0.5 for all plates. **B)** Dose-response assay for the four non-cytotoxic active compounds. Compound effects were tested at 8 different concentrations (μM): 100, 57.1, 32.7, 18.7, 10.7, 6.1, 3.5, 2.0 with 2 replicates per concentration. The Z'-factor determination for plate validation is calculated using the fluorescence anisotropy signals of the controls and reference compound wells. The solid lines represent the mean fluorescence anisotropy value for controls and compounds measurements, the dashed lines denote the average ± 3 SD of the

negative and the positive controls, respectively. **C)** Interference assay for the four non-cytotoxic selected compounds from primary screening. Compound effects were tested at 8 different concentrations (μM): 100, 57.1, 32.7, 18.7, 10.7, 6.1, 3.5, 2.0 with 2 replicates per concentration.
